# An Extremely Rare and Demanding Diagnosis of Primary Renal Synovial Sarcoma: A Case Report

**DOI:** 10.7759/cureus.33954

**Published:** 2023-01-19

**Authors:** Thiago Guimarães, Miguel Cristovão, Nuno Gião, Hugo Pinheiro, Luís Campos Pinheiro

**Affiliations:** 1 Urology, Centro Hospitalar Universitário de Lisboa Central, Lisbon, PRT; 2 Anatomic Pathology, Centro Hospitalar Universitário de Lisboa Central, Lisbon, PRT; 3 Pathologic Anatomy, Centro Hospitalar Universitário de Lisboa Central, Lisbon, PRT

**Keywords:** syt-ssx fusion gene, renal vein tumor thrombus, synovial sarcoma, spindle cell, primary renal synovial sarcoma

## Abstract

Primary renal synovial sarcoma (PRSS) is an extremely rare malignancy. The diagnosis of PRSS is unforeseen due to the absence of clinical and radiological typical aspects. Here, we present a case of a 69-year-old male with complaints of hematuria and left lumbar pain. Abdominal-pelvic computed tomography scan with contrast injection showed a solid mass of 8cm diameter in the left kidney and renal vein tumor thrombus. The patient was further subjected to robotic-assisted left radical nephrectomy and renal vein thrombectomy. We concomitantly performed left adrenalectomy and paraaortic lymphadenectomy. Immunohistochemical and genetic analysis revealed PRSS. This entity is characterized by abnormal chromosomal translocation t(X;18)(p11.2; q11.2) and consequently the characteristic *SYT-SSX* fusion gene. Due to the disease’s rarity and severity, diagnosis and management of PRSS rely upon a demanding and multidisciplinary approach.

## Introduction

Synovial sarcoma (SS) is a soft tissue sarcoma that typically occurs in young adults [1]. This condition accounts for 5-10% of soft tissue sarcoma and usually affects the extremities although several cases have been reported in other locations, rarely in the kidney [1,2]. Primary renal synovial sarcoma (PRSS) can be indistinguishable from other subtypes of renal malignancies by clinical and radiological aspects [3]. Therefore, immunohistochemistry and cytogenetic studies play a key role in the diagnosis of PRSS [1-3]. Herein, we report the case of a 69-year-old male who was diagnosed with PRSS and treated at our hospital.

## Case presentation

**Figure 1:**
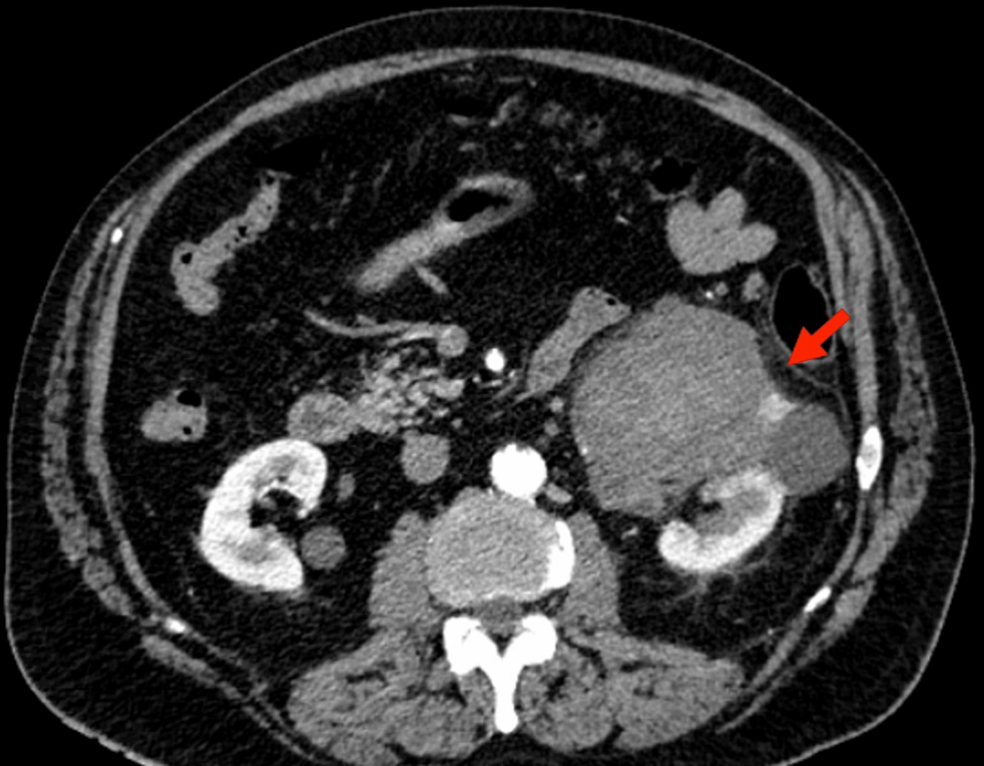
CT scan axial view at presentation CT scan axial view showing a large hypodense mass in the left kidney (red arrow). CT: Computed tomography

**Figure 2:**
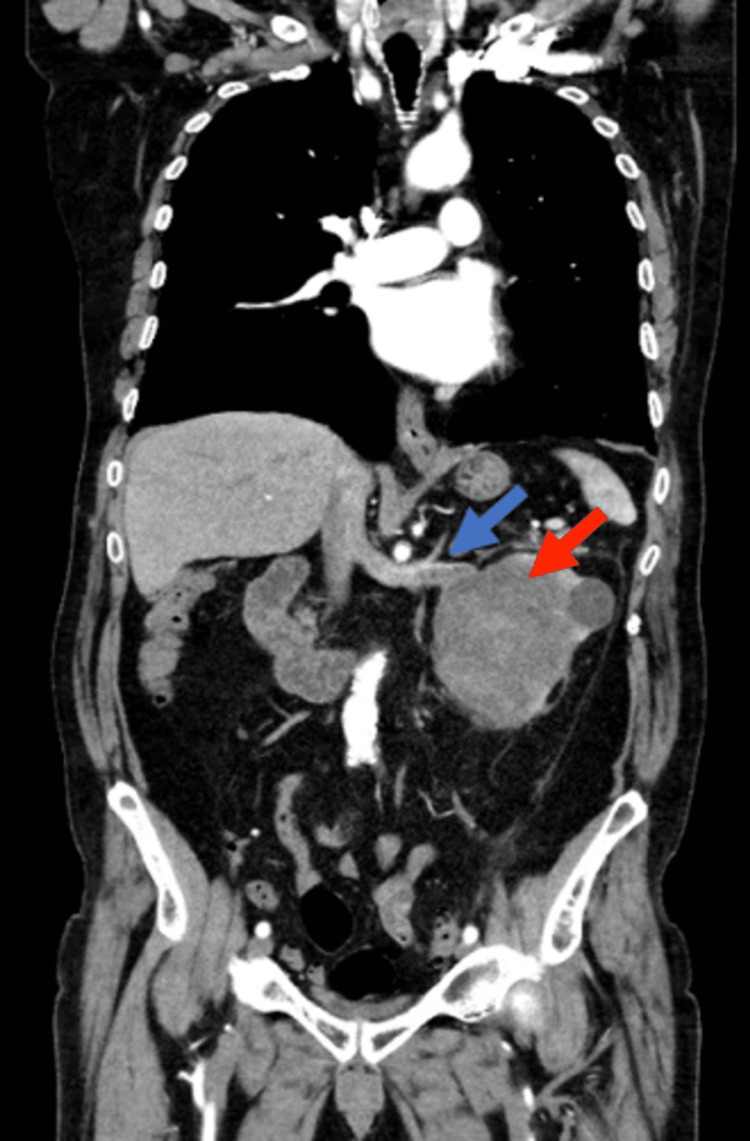
CT scan coronal view at presentation CT scan coronal view showing left renal vein tumor thrombus (blue arrow) and large renal tumor with 8cm diameter (red arrow). CT: Computed tomography

In May 2021, a 69-year-old male presented to our hospital outpatient clinic with complaints of gross hematuria and two-week-long left lumbar pain. His relevant medical history included arterial hypertension, type 2 diabetes, chronic obstructive pulmonary disease, rheumatoid arthritis, and asthma. The patient also had a history of 52-pack-year smoking. However, he had quitted smoking 19 years ago. On physical examination, this patient had a body mass index of 24.5 kg/m^2^. Slight abdominal tenderness more pronounced on the left upper quadrant was detected, but there was no evidence of palpable mass. Hematological investigations showed a hemoglobin level of 15.8 g/dL and urea and creatinine levels of 5.16 mmol/L and 102.55 μmol/L, respectively. The estimated glomerular filtration rate was 64 mL/min/1.73m^2^. Computed tomography (CT) scan imaging with contrast of the abdomen and pelvis showed a large hypodense mass of 8cm diameter in the left kidney and a thrombus in the left renal vein (Figures 1, 2).

Metastatic workup by chest CT showed no evidence of metastasis (clinical TNM: T3a N0 M0). The patient had undergone robotic-assisted left radical nephrectomy through the transperitoneal approach with distal milking of the thrombus to the renal vein and paraaortic lymphadenectomy. Adrenalectomy was also performed. The procedure had no evident complications. The entire operative time was 170 minutes. Estimated patient’s blood loss was 200ml. After surgery, the patient was discharged on the fourth postoperative day. There were no intercurrences in the postoperative evolution.

**Figure 3:**
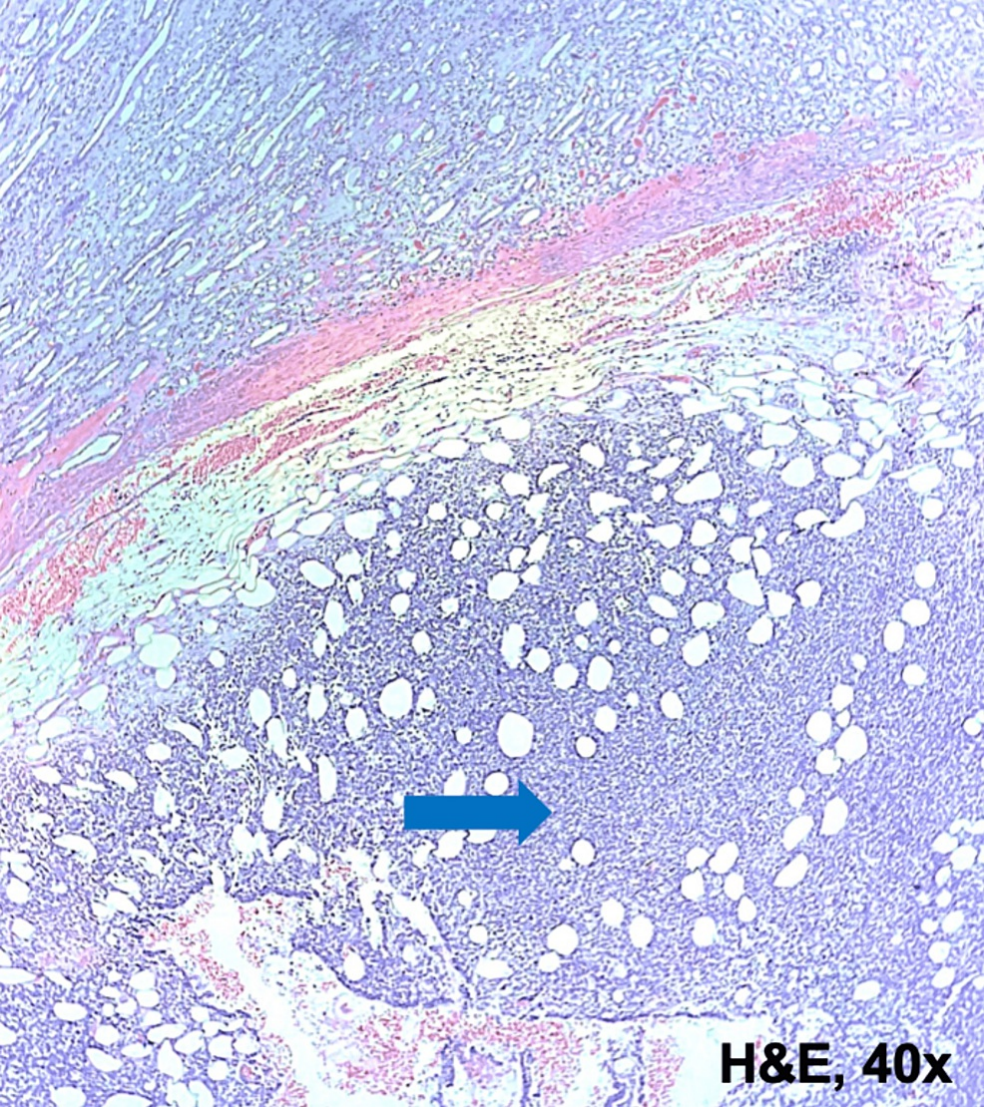
Histopathological findings of primary renal synovial sarcoma Hematoxylin and eosin stain image (40× magnification) showing infiltrative and hypercellular neoplastic proliferation of sheets of small cells (blue arrow).

**Figure 4:**
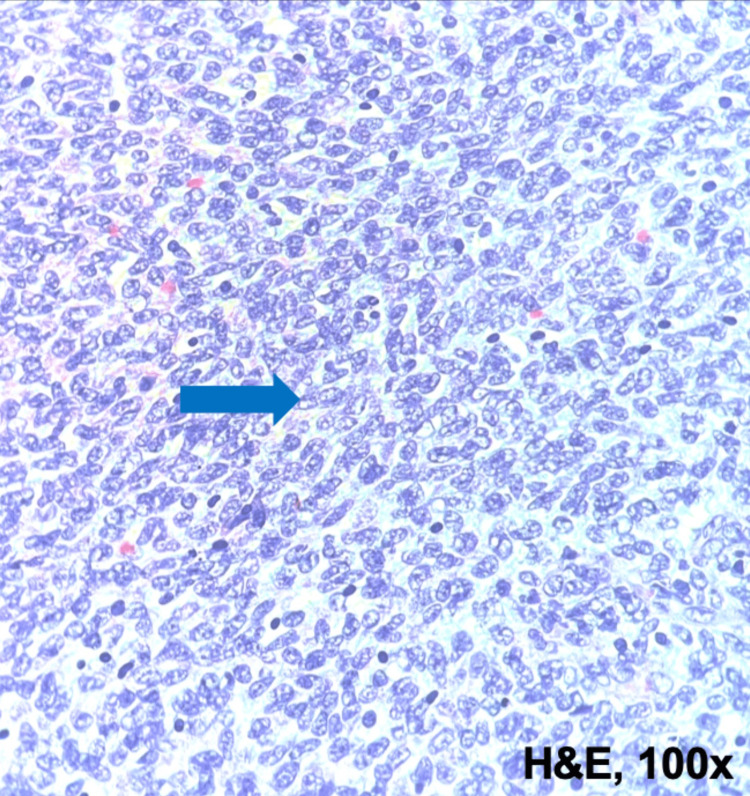
Histopathological findings of primary renal synovial sarcoma. Hematoxylin and eosin stain image (10× magnification) showing sheets of small cells with ovoid, vesicular nuclei, closely overlapping each other, with scant amphophilic cytoplasm (blue arrow).

**Figure 5:**
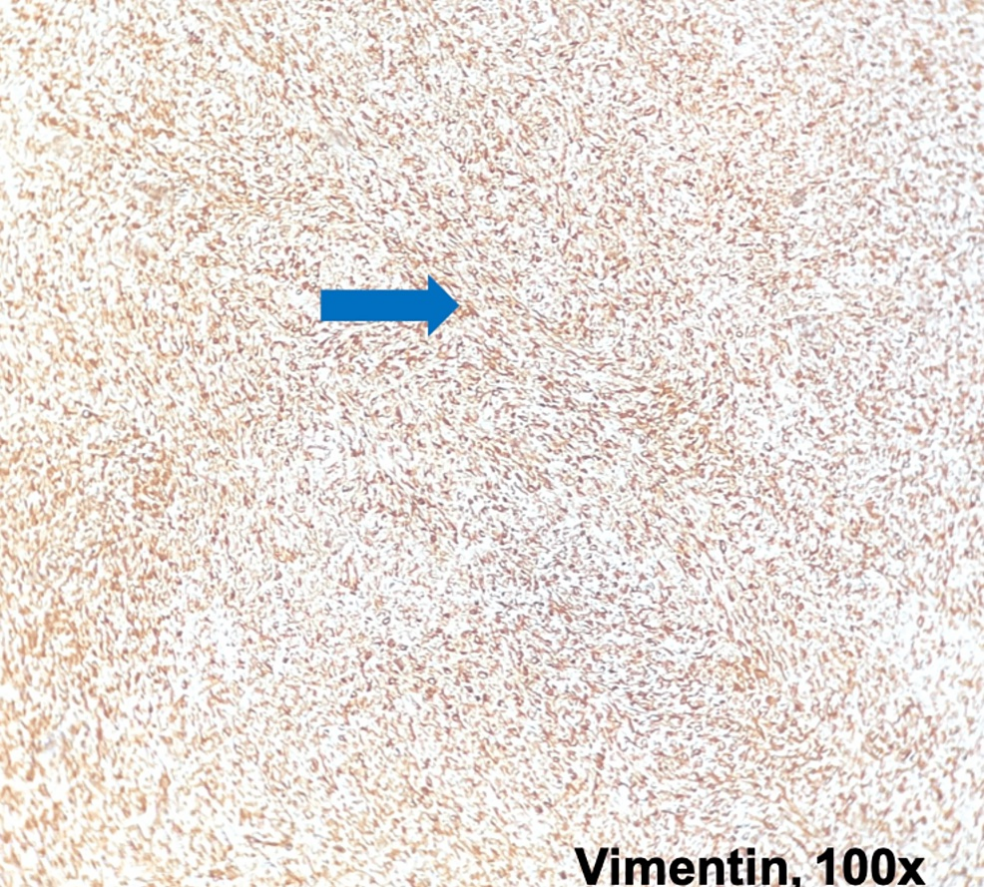
Vimentin expression Immunohistochemistry analysis of renal tissue (100× magnification): the neoplastic proliferation expresses Vimentin diffusely (blue arrow).

**Figure 6:**
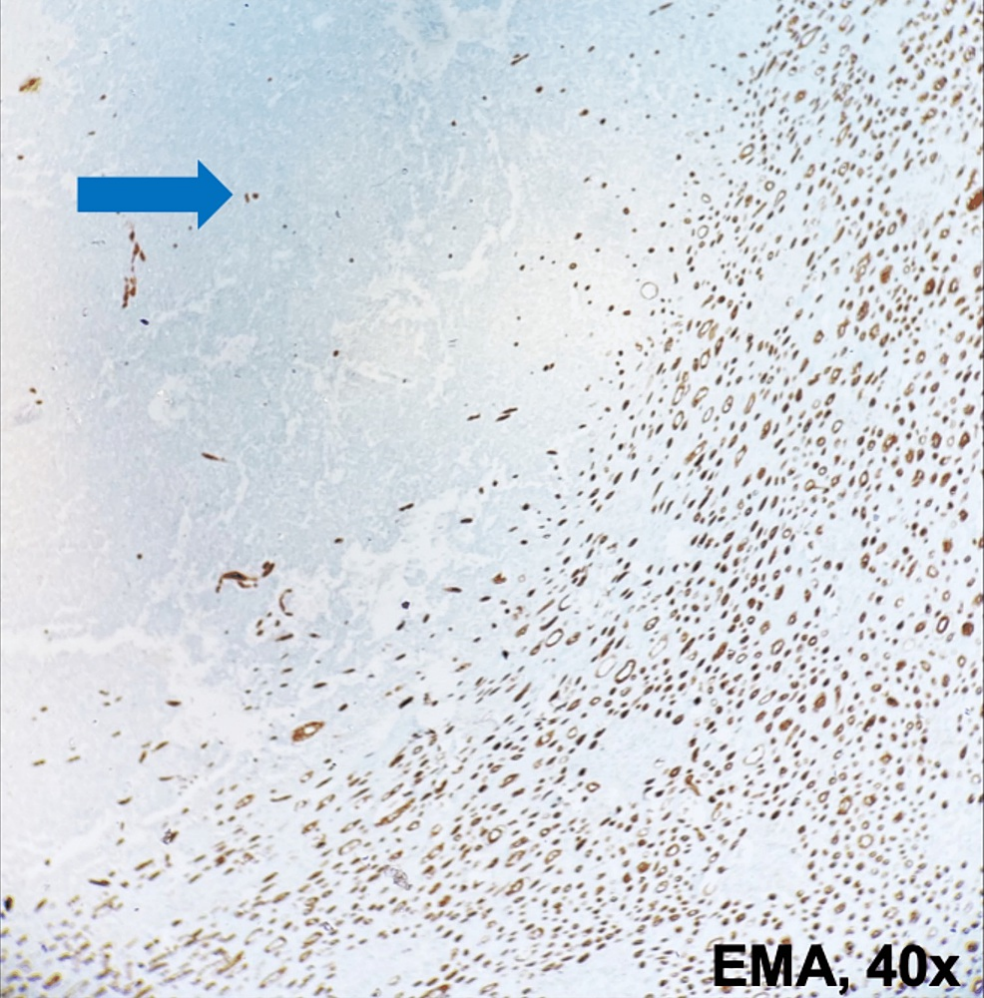
EMA expression Immunohistochemistry analysis of renal tissue (40× magnification): the neoplastic proliferation expresses EMA focally (blue arrow). EMA: Epithelial membrane antigen

Macroscopic examination revealed a solid brownish-white tumor with 8.7cm in greatest dimension. The tumor invaded the pelvicalyceal system and extended to the left renal vein. A tumor thrombus with 2.5cm diameter was detected. Surgical margins were negative. Microscopic examination showed a monomorphic population of spindle-shaped cells with scant cytoplasm, mild atypia, and conspicuous mitotic activity, with a fascicular growth pattern and areas of necrosis (Figures 3, 4). Immunohistochemical evaluation was positive for Vimentin and epithelial membrane antigen (EMA) (Figures 5, 6). The expression of AE1/ AE3 and CAM 5.2 was extremely weak and focal. However, it was negative for S100, chromogranin, synaptophysin, actin, desmin, myogenin, CK7, CD99, WT1, and P63. A definitive diagnosis was achieved by a fluorescence in situ hybridization (FISH) study which revealed structural changes in the SS18 gene (18q11.2). These aspects were compatible with the diagnosis of spindle cell monophasic PRSS.

After surgery, an intensive follow-up protocol was initiated. Chest-abdominal-pelvic CT scan was performed every three months, with no evidence of metastasis until nine months after surgery, where widespread metastatic disease was detected in the liver, pancreas, peritoneum, and lungs. This case was discussed at multidisciplinary evaluation, and doxorubicin was proposed and accepted by the patient. However, after one cycle of doxorubicin 75mg/m^2^, the patient presented progressive worsening of his condition and died. 

## Discussion

Overall, sarcomas of the kidney are diagnosed in 0.8-2.7% of patients with malignant kidney tumors [4]. Leiomyosarcoma is the most diagnosed subtype of kidney sarcoma. It accounts for approximately 40-60% of all kidney sarcomas. Many less frequent subtypes are also rarely reported, including rhabdomyosarcoma, chondrosarcoma, osteosarcoma, liposarcoma, angiosarcoma, hemangiopericytoma [5], and primitive neuroectodermal tumors [6]. PRSS is a rare malignancy. This singular entity was first reported by Argani et al. in 1999 [7]. That report sparked interest and further research throughout the medical community. However, the overall literature remains sparse in this field. Currently, approximately only 200 cases of PRSS have been described [8,9]. More recently, Blas et al. provided in a systematic review, a precise summary of the clinical characteristics and treatment of patients presenting with PRSS [8,9]. In that study, the mean age of diagnosis was 38.6 years, and PRSS was slightly more frequent in men [8]. Abdominal pain and hematuria are the most frequent symptoms at presentation [2,9]. Lymphadenopathies are not common findings in PRSS [8].

The absence of pathognomonic findings makes the diagnosis of PRSS demanding. However, CT scan and magnetic resonance imaging (MRI) may provide useful information. In a retrospective study with five patients carried out by Lv et al., either a well-defined solid-cystic mass with heterogeneous contrast enhancement or a cystic lesion with septal enhancement can be observed [10]. Additionally, the “rapid wash-in and slow wash-out’ pattern of contrast enhancement of the solid constituent may help to discriminate between SS and more usual variants of renal cell carcinoma [10]. MRI plays an essential role in the diagnosis of soft tissue sarcomas [11]. MRI findings of PRSS were first described by Zakhary et al [12]. Those findings show a well-defined nodular heterogeneous lesion larger than 5cm with a hypointense signal on T1WI (T1-weighted-image) and also areas of hemorrhage and necrosis. ‘Triple sign’ on T2WI (T2-weighted-image) was also observed and refers to areas of low, intermediate, and high signal intensity [12]. According to Blas et al., 29 patients of 185 published cases with PRSS presented with thrombus [9]. Thrombus extension to the renal vein was described in 34.5% of patients (10/29).

Histologically, PRSS classification is similar to SS in other locations. Macroscopically, this tumor presents as a large solid mass with areas of cystic changes, hemorrhage, and necrosis [8]. At a microscopic level, SS is further characterized into three histologic subtypes: monophasic, biphasic, and poorly differentiated. The monophasic variant is the most common (50-60%) [13]. Monophasic SS is characterized by ovoid-spindle shape morphology. The biphasic variant is composed of both spindle and epithelial components. It represents 20-30% of lesions [13]. The poorly differentiated variant is composed of uniform, densely packed small ovoid blue cells. This variant represents up to 15%-25% of all SS [13]. The pathological differentiation from other spindle cell malignancies of the kidney such as adult Wilms tumors and primary PNET/Ewing, sarcomatoid renal cell carcinoma, and undifferentiated carcinoma is challenging [3,8]. Immunohistochemical and genetic analysis play an important role in the diagnosis of SS [3,8,9]. Immunopositivity for vimentin, CD99, and BCL2 of the spindle cells and that of cyst epithelium for cytokeratins (ex.: EMA and CK7) aid in diagnoses of SS [13,14]. It would be relevant to add TLE1 since it is the most sensitive and specific immunohistochemical marker [15]. However, this test was not available at our institution. Since immunohistochemistry results were not enough to achieve the diagnosis, cytogenetics and molecular studies were used. FISH or RT-PCR studies often reveal an abnormal chromosomal translocation t(X;18)(p11.2;q11.2) and SYT-SSX gene fusion proteins, respectively [2]. Those genetic patterns are found in at least 90% of the SS cases, and those are very accurate for the diagnosis of SS [2,14].

Patients diagnosed with PRSS should be referred to a tertiary center with an experienced multidisciplinary team specialized in soft tissue sarcoma [16]. Due to its rarity, the treatment choices are also in debate. Although no standard treatment has been approved, radical nephrectomy is considered the treatment of choice to achieve local control [9]. Adjuvant chemotherapy was performed in approximately 1/3 of published cases, with a preference for the administration of ifosfamide-based chemotherapy and doxorubicin or epirubicin [9]. According to previous studies, PRRS has an aggressive course with an increased metastasis potential and poor survival outcome [8,9,17]. According to Iacovelli et al., the median disease-free survival of PRSS patients was 33 months with a short life expectancy of six months when there is metastatic disease [17]. 

## Conclusions

PRSS is an extremely rare entity. Considering clinical and radiologic features, PRSS is not easily distinguishable from other renal tumors. Typical aspects of immunohistochemical and cytogenetics evaluation demonstrating an abnormal chromosomal translocation t(X;18)(p11.2;q11.2) and SYT-SSX gene fusion proteins are key for diagnosis. Radical nephrectomy is the treatment of choice. Adjuvant chemotherapy should be only considered in selected patients since there are few reporters available in the literature. Therefore, more studies are needed to better understand the role of adjuvant treatments. Multidisciplinary evaluation is recommended in order to ensure the optimal health care plan for the patient. However, the prognosis remains fairly poor.

## References

[1] Gazendam AM, Popovic S, Munir S, Parasu N, Wilson D, Ghert M (2021). Synovial Sarcoma: a clinical review. Curr Oncol.

[2] Paláu L MA, Thu Pham T, Barnard N, Merino MJ (2007). Primary synovial sarcoma of the kidney with rhabdoid features. Int J Surg Pathol.

[3] Lopes H, Pereira CA, Zucca LE, Serrano SV, Silva SR, Camparoto ML, Cárcano FM (2013). Primary monophasic synovial sarcoma of the kidney: a case report and review of literature. Clin Med Insights Oncol.

[4] Öztürk H (2015). Prognostic features of renal sarcomas (Review). Oncol Lett.

[5] Grampurohit VU, Myageri A, Rao RV (2011). Primary renal synovial sarcoma. Urol Ann.

[6] Uhlig J, Uhlig A, Deshpande HA, Hurwitz ME, Humphrey P, Kim K (2021). Renal sarcomas: epidemiology, treatment and outcomes. J Clin Oncol.

[7] Argani P, Faria PA, Epstein JI, Reuter VE, Perlman EJ, Beckwith JB, Ladanyi M (2000). Primary renal synovial sarcoma: molecular and morphologic delineation of an entity previously included among embryonal sarcomas of the kidney. Am J Surg Pathol.

[8] Mastoraki A, Schizas D, Karavolia DM (2022). Primary synovial sarcoma of the kidney: diagnostic approach and therapeutic modalities for a rare nosological entity. J Pers Med.

[9] Blas L, Roberti J (2021). Primary renal synovial sarcoma and clinical and pathological findings: a systematic review. Curr Urol Rep.

[10] Lv XF, Qiu YW, Han LJ (2015). Primary renal synovial sarcoma: computed tomography imaging findings. Acta Radiol.

[11] Scalas G, Parmeggiani A, Martella C (2021). Magnetic resonance imaging of soft tissue sarcoma: features related to prognosis. Eur J Orthop Surg Traumatol.

[12] Zakhary MM, Elsayes KM, Platt JF, Francis IR (2008). Magnetic resonance imaging features of renal synovial sarcoma: a case report. Cancer Imaging.

[13] Murphey MD, Gibson MS, Jennings BT, Crespo-Rodríguez AM, Fanburg-Smith J, Gajewski DA (2006). From the archives of the AFIP: imaging of synovial sarcoma with radiologic-pathologic correlation. Radiographics.

[14] Katabathina VS, Vikram R, Nagar AM, Tamboli P, Menias CO, Prasad SR (2010). Mesenchymal neoplasms of the kidney in adults: imaging spectrum with radiologic-pathologic correlation. Radiographics.

[15] Duran-Moreno J, Kampoli K, Kapetanakis EI (2019). Pericardial synovial sarcoma: case report, literature review and pooled analysis. In Vivo.

[16] Nystrom LM, Reimer NB, Reith JD, Dang L, Zlotecki RA, Scarborough MT, Gibbs CP Jr (2013). Multidisciplinary management of soft tissue sarcoma. ScientificWorldJournal.

[17] Iacovelli R, Altavilla A, Ciardi A, Urbano F, Manai C, Gentile V, Cortesi E (2012). Clinical and pathological features of primary renal synovial sarcoma: analysis of 64 cases from 11 years of medical literature. BJU Int.

